# Prenatal PCB Exposure and Thymus Size at Birth in Neonates in Eastern Slovakia

**DOI:** 10.1289/ehp.9769

**Published:** 2007-08-14

**Authors:** Hye-Youn Park, Irva Hertz-Picciotto, Jan Petrik, Lubica Palkovicova, Anton Kocan, Tomas Trnovec

**Affiliations:** 1 Division of Epidemiology, Department of Public Health Sciences, School of Medicine, University of California, Davis, California, USA; 2 Department of Toxic Organic Pollutants and; 3 Department of Environmental Medicine, Slovak Medical University, Bratislava, Slovakia

**Keywords:** *p,p′*-DDE, *p,p′*-DDT, PCBs, thymic index, immune status, prenatal

## Abstract

**Background:**

Polychlorinated biphenyls (PCBs) are ubiquitous environmental toxicants, for which animal studies demonstrate immunotoxic effects, including thymic atrophy and suppressed immune responses; human investigations of similar end points are sparse. The thymus is essential for the differentiation and maturation of T-cell lymphocytes.

**Objectives:**

The objective of this study was to examine the association between prenatal PCB exposures and estimated thymus volume in infants from eastern Slovakia, a region where PCBs were produced until 1984.

**Methods:**

Mothers were enrolled at delivery, and maternal blood samples were collected for analysis of 15 PCB congeners, *p,p*′-DDT [1,1,1-trichloro-2,2′-bis(*p*-chlorophenyl)ethane], and *p,p*′-DDE [1,1-dichloro-2,2-bis(*p*-chlorophenyl)ethylene]. Each mother was interviewed to obtain information on sociodemographic characteristics, past pregnancies, occupational history, medication history, and living environment. Neonatal thymus volume was estimated using ultrasound measurements on the third or fourth day after birth. Thymic index was calculated on 982 newborns from mothers with PCB measurements. We developed a predictive model of the natural log of the thymic index using multiple linear regression with covariates selected from the bivariate analyses.

**Results:**

Prenatal PCB exposure was associated with a smaller thymic index at birth [β= −36 (natural log-transformed; nanograms per gram lipids); *p* = 0.047]. District of residence and delivery also predicted thymic index. Male sex, later gestational age, larger birth weight *z*-score, and Roma ethnicity were associated with a larger thymic index, whereas respiratory illness was associated with a lower thymic index.

**Conclusions:**

This study provides the first evidence to date that PCB exposure in neonates is associated with a smaller thymic volume, suggesting possible impaired immunologic development.

Polychlorinated biphenyls (PCBs), a class of 209 congeners with two linked phenyl rings and variable chlorination in different sites, were introduced in 1929. They were widely used as dielectric fluids in electrical transformers and capacitors, and as heat exchangers or hydraulic fluids. Improper disposal has been a major means by which PCBs enter the environment. These chemicals are persistent in the environment and the human body because of their high lipophilicity and stability. Their production and use were banned in most industrialized countries in the late 1970s because of toxicity to wildlife (Elliott et al. 2000; Ross et al. 2004).

PCBs have shown toxic effects on various organs including tissue of the nervous, reproductive, and immunologic systems. Suppressed immune function can lead to increased susceptibility to infectious disease or certain types of cancers. A study in the Netherlands showed that prenatal or lactational exposures to PCBs were associated with changes in immune profiles in children (Weisglas-Kuperus et al. 1995, 2000).

The thymus is essential for the differentiation and maturation of T lymphocytes. Administration of PCBs in rodents, specifically dioxin-like PCBs, *in vivo* and *in vitro* induces thymic atrophy and immune suppression. In particular, thymic atrophy has been observed consistently in studies using various species after *in vivo* exposure to PCBs or dioxin (Beineke et al. 2005; DeCaprio et al. 1986; Goff et al. 2005; Silkworth and Antrim 1985). Some authors have suggested these effects might result from activation of the aryl hydrocarbon (Ah) receptor (Shimada 1987; Silkworth et al. 1986; Tryphonas et al. 2001). Of all tissues in the rat, the thymus has one of the highest expression levels of Ah-receptor mRNA (Carver et al. 1994). Other studies indicate that 2,3,7,8-tetrachlorodibenzo-*p*-dioxin (TCDD) or non-coplanar PCBs might induce immunotoxic effects through Ah-receptor independent mechanisms (Kerkvliet et al. 1990; Tryphonas et al. 2001). However, the mechanisms for immunotoxicity by PCBs are not fully understood, and given a broad range of interaction between the neurologic and immune systems, organs other than the thymus may be involved.

The thymic index, derived from sonographic measurements, has been used as an approximation of thymus size and is regarded as reliable, especially in young children (Hasselbalch et al. 1996). The thymic index is highly correlated with the actual volume and weight of the thymus (Hasselbalch et al. 1996). Previous studies have documented the development of the thymus in humans: The size and the thymic function increase to a maximum in infants (George and Ritter 1996; Steinmann et al. 1985) and then gradually decrease beginning at about 8 months (Hasselbalch et al. 1997). Some reports found breast-feeding, birth weight, birth length, and head circumference to be positively associated with thymic index (Benn et al. 2001; Hasselbalch et al. 1997; Iscan et al. 2000; Yekeler et al. 2004). Malnutrition, infection, short gestation, stress, and zinc depletion have been associated with a smaller thymic index (Dominguez-Gerpe and Rey-Mendez 2003; Jeppesen et al. 2003; Malpuech-Brugere 2001). However, we found no previous study that has directly addressed the thymic index as an outcome in humans in relation to hazardous chemical exposures in the environment. In this project, we studied thymus size in neonates as an indicator of dysregulated immunologic development in relation to prenatal exposure to PCBs.

## Materials and Methods

### Study population

Mothers were recruited for this study at the time of delivery from two districts in eastern Slovakia during 2002–2004: Michalovce, with high PCB contamination from a chemical manufacturing plant; and Svidnik, located around 70 km to the northwest, with substantially lower levels of PCBs. Each district has only one hospital, and the vast majority of births from these regions occur in these hospitals. For eligibility criteria, we excluded *a*) mothers with more than four previous births, *b*) mothers < 18 years of age, *c*) mothers who had resided < 5 years in their district, *d*) mothers with a major illness during pregnancy, and *e*) infants who had severe birth defects. The study subjects gave written informed consent. Of 1,134 eligible participants, PCB measurements in maternal serum were available on 1,076. Thymic index data from 982 were available after excluding subjects lacking thymic index measurements (*n* = 50), or radiologist information (*n* = 16), or who had implausible values of thymic index (*n* = 28). This study was approved by institutional review boards of the University of California, Davis, and the Slovak Medical University.

### Specimen collection

After delivery, maternal blood specimens (30–35 mL) were collected by a nurse using venipuncture into heparinized, standard, and EDTA vacutainers. All tubes were refrigerated at 5–10°C immediately after collection of blood. Samples were transported to the Department of Biochemistry of each local hospital within 2 hr for processing. Serum was isolated by centrifugation (15 min at 3,000 rpm). Three milliliters of serum were placed into an 8-mL glass vial labeled with the identification code and stored frozen at −18°C. These were transported to the Slovak Medical University in Bratislava in thermo boxes with cooling cartridges to prevent thawing, and stored at −18°C for PCB analysis; a small amount (0.2 mL) was aliquoted for lipid measurement and stored.

### PCB measurement

We determined the concentrations of 15 congeners of PCBs [PCB International Union of Pure and Applied Chemistry (IUPAC) nos. 28, 52, 101, 105, 114, 118, 123, 138, 153, 156, 157, 167, 170, 180, and 189), *p,p*′-DDT [1,1,1-trichloro-2,2′-bis(*p*-chlorophenyl)ethane], and *p,p*′-DDE [*p,p*′-DDE (1,1-dichloro-2,2-bis(*p*-chlorophenyl)ethylene] in the maternal serum samples by high-resolution gas chromatography (GC) with electron capture detection (Conka et al. 2005; Kocan et al. 1994). Briefly, the procedure included standardized extraction, cleanup, and quantification. PCB-174 as an extraction standard had been added to the blood serum before the analytes were isolated using solid phase extraction. The SPE extract was concentrated and then cleaned by passing through a Florisil-H_2_SO_4_/silica gel column. The eluate was then evaporated to a small volume, and PCB-103 was added as a syringe standard to correct the final volume of samples before GC injection. An aliquot of the mixture was injected and analyzed on a chromatography system (HP 5890; Hewlett-Packard, Palo Alto, CA, USA) equipped with a Ni-63 electron capture detector using a 60-m DBP-5 capillary column (J&W Scientific, Folsom, MA, USA). Quantification was based on the calibration curve generated by authentic PCB standard solutions at five different concentration levels. Quality control activities consisted of analyses of samples in batches of 10 simultaneously with a blank sample and in-house reference material (spiked porcine serum). Response for a particular congener had to be in the range of 90–110% using the concentration of the middle point of the calibration curves for that congener. We determined the limit of detection for each analyte as the mean of background noise plus 3 standard deviations from five reagent blank samples.

Six of the individual PCB congeners were selected to be included into PCB sum (PCB IUPAC nos. 118, 138, 153, 156, 170, and 180) based on having < 20% of samples below the limit of detection (LOD). When one of these PCB congeners was below the limit of detection in an individual’s sample, we imputed by assigning the LOD value divided by the square root of 2 (Persky et al. 2001; Weisskopf et al. 2005). PCB-sum concentrations were determined on a wet weight basis (nanograms per milliliter); subsequently these PCB concentrations were adjusted for lipids (nanograms per gram).

The Department of Toxic Organic Pollutants at the Slovak Medical University in Bratislava (SMU) performed the laboratory analyses. The laboratory has participated in intercalibration studies organized by the World Health Organization (2000) and the German Agency for Occupational and Environmental Medicine (Deutsche Gesellschaft fuer Arbeitsmedizin und Umweltmedizin e.V.).

### Lipids measurement

We estimated total serum lipids (TL) using the enzymatic summation method (Akins et al. 1989). We measured serum total cholesterol (TC) and triglyceride (TG) using a DuPont Automatic Clinical Analyzer III analyzer (Dupont, Jonesboro, AR, USA), and cholesterol oxidase without cholesterol esterase was used to detect free cholesterol (FC). The method by Takayama et al. (1977) was used to determine serum choline-containing phospholipids (PL). Total serum lipids were calculated from the formula


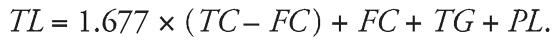


The lipids were measured at a biochemical laboratory accredited by the Slovak National Accreditation Service located at the Ministry of Defense Military hospital in Bratislava.

### Thymus measurement

The thymus was measured on the 3rd or 4th day after birth in 982 neonates from October 2002 through December 2004. Four radiologists from Michalovce and two radiologists from Svidnik measured the thymus using a sonographic scanner [in Michalovce: Esaote 580 FD Caris plus (convex probe 7.5 MHz); Esaote SpA, Genova, Italy; in Svidnik: Esaote AU 5 Harmonic (convex probe 7.5 MHz); Esaote SpA, Firenze, Italy]. The thymus is located in the anterior mediastinum in front of the large vessels, and is well delineated by sonography. The infant was examined on his or her back with the neck extended and head fixed in an optimal position for measurement. A transsternal approach was used to measure the maximal transverse diameter (width) of the thymus. In the plane perpendicular to this width, the largest sagittal area (longitudinal scan plan) was also measured. These two measurements were multiplied to obtain the thymic index. The thymic index was used as an estimate of the thymus volume.

### Data collection

The two major data sources for covariates were an interview with the mother conducted by trained staff during the 5-day hospital stay, and the newborn medical record. The interview obtained information on sociodemographic characteristics, past pregnancies, occupational history, medical conditions, medication history before and during the pregnancy, and living environment. Romani ethnicity was attributed to the mother if the ethnic origin of either of her parents was Romani, the Romani language was spoken at the home of the child, or the mother was planning to raise her child with the Romani language. This definition of Romani was subsequently independently confirmed by a Slovak member of the research team who used additional information such as the family’s last name to confirm the ethnicity. Ethnicity was categorized into two groups: Slovakian/other eastern European or Romani. Smoking history during or before pregnancy was extracted from the interview data. Alcohol consumption was defined as one or more drinks of beer, wine, or liquor per week during pregnancy or in the 3 months before pregnancy. Maternal illness history included respiratory infection, asthma, or allergy during the same time period. Parity was coded as an ordinal variable (0–4).

The abstraction of the newborn’s medical records included birth weight and gestational age (weeks). The estimate of gestational age was based on last menstrual period (LMP) reported in the medical records and the clinical judgment made by the woman’s physician.

### Data analysis

Univariate distributions were examined and implausible values were investigated and resolved by communication with field staff. The thymic index was highly skewed (test of normality by Shapiro-Wilk, *p* < 0.0001), so we used the logarithmic transformation (log_e_) of the thymic index in the analysis as the outcome variable. As the primary predictor variable of interest, the total maternal PCB concentration based on the six most abundant congeners was adjusted for serum lipid concentration and expressed as nanograms per milligram (equivalent to micrograms per gram) total lipid. Because the PCB distribution was also skewed (*p* < 0.0001), these values were log-transformed as well to reduce the influence of extreme values. Birth weight was expressed as a *Z*-score standardized for sex, parity, and gestational age based on all births in Slovakia in 2004.

As a first step in the data analysis, we conducted simple linear regressions (bivariate analyses) with the thymic index as the outcome. Possible confounders such as sex, socioeconomic status (parental education), smoking, alcohol consumption, ethnicity, and maternal medical history including such ailments as diabetes, hypertension, hypothyroidism, allergy, asthma, and respiratory disease were evaluated. Then a multiple linear regression model was developed with covariates selected from the bivariate analyses. Variables with low or no association with the thymic index at *p* > 0.3 were excluded from final multiple linear regression models. We used a backward selection approach to develop a final predictive model. After exponentiation from the final regression model, expected thymic indices were calculated; results are presented as percent change in thymic index across the interquartile range and for the 10th–90th increase in PCBs respectively.

**Figure 1:**
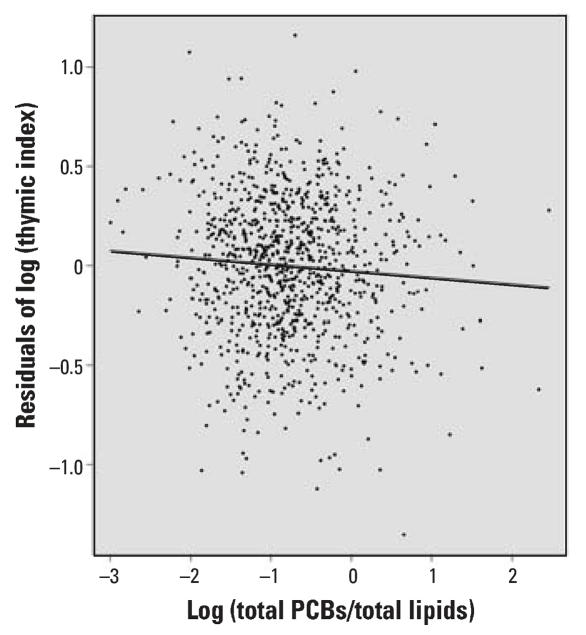
Scatterplot of residuals for thymic index in relation to log maternal serum PCBs after adjusting for other predictors.

Scatterplots of residuals (the difference between the observed thymic index values and those predicted by the regression equation) for thymic index were plotted from the multivariate model that included all variables in the final model except PCBs. Figure 1 shows the partial residuals from a model that included only the covariates as predictors, as well as the best-fitting line after inclusion of log-transformed PCBs, demonstrating the decreasing trend of the log-transformed thymic index with increasing exposure. The SAS statistical package was used for analysis (version 9.1; SAS Institute Inc., Cary, NC, USA).

## Results

Table 1 describes characteristics of the cohort. About one-fifth of the mothers was > 30 years of age and around 7% had a university degree. In this study population, 92% of participants were married or living with their partners, 36% were smokers, and 30% consumed alcohol during either the pregnancy or the 3 months before conception. 59% of women were multiparous and 8% had more than two previous children. One percent and 4% of mothers reported asthma and allergy, respectively, and 20% reported respiratory infections during the period from 3 months before conception through the end of pregnancy.

The median thymic index was 8.7 (mean, 9.6; range, 2.1–32.5), and 97% were < 20. The distribution of thymic index was skewed to the right. Table 2 reports the information on thymic index from six radiologists in two districts. The measurements of radiologists 5 and 6, both from Svidnik area, seemed to be larger and had wider variation than those of radiologists from Michalovce. Radiologist 5 was the most experienced of all of them.

The mean, median, and 10th and 90th percentiles of the PCB sum were 620, 440, 190, and 1,170 ng/g serum lipids respectively. Table 3 shows the final multiple regression model predicting the log thymic index as a function of the lipid-adjusted PCB sum, adjusting for sex of infant, maternal smoking and alcohol history, Romani ethnicity, gestational age, *Z*-score of birth weight, district of residence, and maternal respiratory infection history during pregnancy or the 3 months before pregnancy. Higher serum PCB concentrations were associated with a smaller thymic index. For an increase in PCB across the interquartile range [280–700 ng/g (ug/g) serum lipids], the thymic index decreased by 3%, (*p* = 0.047). An increase from the 10th to 90th percentile [190–1,170 ng/g serum lipids] was associated with a 7% reduced thymic index. Female infants had a smaller thymic index by 10% in comparison with male infants. Maternal smoking, alcohol, and respiratory infection histories were associated with a smaller thymic index by 3%, 5%, and 6% respectively; however, smoking and alcohol consumption were not statistically significant. The district differences observed in the crude data were upheld after adjusting for covariates. Each unit of *Z*-score of birth weight adjusted for gestational age (1 SD increase) and each week of gestational age increased thymic index by 13% and 3%, respectively.

We tested *p,p*′-DDE and *p,p*′-DDT in the multiple linear regression models. Although the same direction of effect was observed as was seen in PCBs, the coefficients were small and not significant (results not shown).

Table 4 presents a separate final multiple linear regression model in which we focused on the measurements (*n* = 187) from the radiologist who is the most experienced. Applying the beta coefficient from this model to the population interquartile range of PCBs yielded a decrease in the thymic index of 8.9% (*p* = 0.033). If PCBs increased from the 10th to the 90th percentile, the thymic index was reduced by 16.9%.

Figure 1 shows the scatterplot of the log-transformed maternal serum PCBs against the residuals of the log thymic index from a multiple linear regression model with all predictor variables except PCBs. This plot visually presents the degree of negative slope explained by PCBs alone.

## Discussion

The thymus is a flat, bi-lobed organ located above the heart. It plays a critical role in the differentiation and maturation of T-cell lymphocytes in immune system. These T lymphocytes are mainly responsible for cell-mediated immunity, which does not involve antibodies but protects against cancer cells, intracellular bacteria, and viruses. T lymphocytes secrete cytokines that can contribute to activating other immune cells or can serve a cytotoxic or regulatory function (Goldsby et al. 2003).

It has been reported that certain risk factors such as malnutrition, zinc depletion, stress, HIV or other infection, preterm birth, and seasonality [e.g., birth during hungry season, July–December, in Gambia (Collinson et al. 2003)] are associated with a smaller thymus size (Collinson et al. 2003; Dominguez-Gerpe and Rey-Mendez 2003; Jeppesen et al. 2003; Malpuech-Brugere 2001). Information is more limited on possible mechanisms to support these reported associations. Suggested hypotheses (Dominguez-Gerpe and Rey-Mendez 2003) include enhanced apoptosis in thymocytes, or reduced migration of precursor T lymphocytes from bone marrow to thymus. A small thymic index at birth was associated with reduced interleukin-7 in breast milk of the mother.

*In vivo* rodent experiments and *in vitro* studies have shown loss of thymocytes or thymic atrophy induced by certain PCB congeners (Beineke et al. 2005; Robertson et al. 1984; Tan et al. 2003). Other studies suggest this occurs by enhanced differentiation with impaired proliferation of thymocytes (Esser et al. 1994; Kremer et al. 1994; Lai et al. 1994). PCBs may interfere with the interaction of stromal cells that interact with the developing thymocytes rather than acting directly on thymocytes themselves (Esser et al. 1994; Kremer et al. 1994; Lai et al. 1994). Thymocytes develop in a three-dimensional stromal-cell network that consists of epithelial cells, dendritic cells, and macrophages (Goldsby et al. 2003). The interaction between stromal cells and thymocytes leads to proliferation and maturation of a repertoire of T cells (Van 1991). Fine et al. (1989, 1990) reported from an *in vivo* study with BALB/c mice that TCDD, chemical compounds structurally similar to PCBs, caused thymic atrophy through changes in lymphocyte precursors in the bone marrow and in the fetal liver. Camacho et al. (2004) showed that in C57BL/6 mice after perinatal exposure, TCDD induced increased apoptosis causing thymic atrophy.

Before sonographic examination became available, thymus size measurements were possible only on autopsy. Several earlier studies using sonographic measurements were either case reports or used their own estimates of thymus size such as thickness. Since Hasselbalch et al. (1996) suggested the thymic index as an estimate of the size of thymus, this approach has been adopted by several researchers (Aaby et al. 2002; Iscan et al. 2000; Jeppesen et al. 2003; Yekeler et al. 2004).

Our findings are consistent with previous studies showing the thymic index to be positively correlated with birth weight (Hasselbalch et al. 1997; Iscan et al. 2000; Yekeler et al. 2004) and gestational age (Jeppesen et al. 2003). Male infants had larger thymic index even after adjustment for birth weight in our study; Aaby et al. (2002) also reported a larger thymic index in boys, though this study did not adjust for birth weight. We also observed that maternal smoking, alcohol consumption, and respiratory infections during pregnancy or the 3 months before conception were associated with a lower thymic index, although smoking and alcohol consumption were not statistically significant in our data.

We found increased thymic index among infants with Romani ethnicity. Considering the deficient nutrition (e.g., the estimated average daily intake of vitamin C is 44% of recommended daily allowance) (Brazdova et al. 1998); social conditions for the Romani in eastern Slovakia including low socioeconomic status, slum housing, and suboptimal hygienic conditions; and a high prevalence of smoking and alcohol consumption (Brazdova et al. 1998; Koupilova et al. 2001; Krajcovicova-Kudlackova et al. 2004; Vivian and Dundes 2004), a reduced thymic index might have been expected. Because we adjusted for smoking and alcohol consumption (as well as birth weight, which is also lower in Romani children), the mechanism leading to increased thymic index remains unclear. Interestingly, in our data, the Romani group showed lower prevalence, compared with non-Romani, of maternal illness history such as respiratory infections (17.2 vs. 21.3%), asthma (0.5 vs. 1.1%), or allergy (0 vs. 4.6%) during pregnancy or in the 3 months before pregnancy. Therefore, the possibility of selection bias (e.g., participation of more healthy subjects from Romani background) cannot be excluded in this study. *In utero* exposures to unmeasured environmental factors/pollutants during critical windows of gestation in the Romani ethnic group might play a role in the increased thymic index as well.

Despite the long time period since their arrival to Europe from Asia, the Romani maintain significantly smaller stature in terms of body height and weight and percentage of body fat, and a trend toward lower concentrations of the thyroid hormone thyroxine (Ginter et al. 2001); thus genetic differences cannot be excluded. Olesen et al. (2005) observed a larger thymic index was associated with children 0–6 years of age having atopic dermatitis compared with healthy children, and suggested that this positive association might be related to an unbalanced establishment of the peripheral T-lymphocyte system.

Six radiologists measured the thymic index from the two districts where different instruments were used. Inter- or intrareliability comparisons among these six were not performed. Inclusion of a set of indicator variables for radiologist in the final model did not influence the parameter estimates for PCBs or for other covariates. We also tested models for the PCB association with thymic index within radiologists, and our finding was most strongly confirmed with infants examined by the most experienced radiologist (no. 5, from Svidnik).

*p,p*′-DDE and *p,p*′-DDT were also evaluated in the multiple linear regression models, but were not significant. When we summed PCBs, *p,p*′-DDT, and *p,p*′-DDE, the parameter estimate was very close to that for PCBs alone.

Zinc is an essential trace mineral in humans and is known as an inhibitor of apoptosis. Zinc deficiency is associated with thymic atrophy and lymphopenia (Fraker et al. 2000), and zinc supplements resulted in faster recovery of thymus tissue in malnourished children (Chevalier et al. 1996). Venkataraman et al. (2004a, 2004b) found that serum zinc concentration was significantly reduced in PCB-exposed rats. A possible mechanism for the observed reduced thymic size in the present study might be zinc deficiency induced by PCB exposure.

Our results provide the first evidence to date that higher prenatal PCB exposures are associated with a smaller thymic index at birth, which may imply impaired or delayed immune development *in utero*. Other researchers have noted that PCBs can alter immunologic function. Weisglas-Kuperus et al. (1995, 2000) reported increased numbers of CD8^+^ T lymphocytes and lower antibody tiers in response to rubella and mumps vaccines in association with higher prenatal PCB exposure in infants. Also, they observed that significant recurrent otitis media and higher prevalence of chicken pox were associated with a higher PCB body burden at 42 months of age. The median level of the sum of PCBs 118, 138, 153, and 180 in the Dutch study (Weisglas-Kuperus et al. 1995, 2000) was 2.07 μg/L, whereas in our study population it was 3.67 μg/L. If we compare the median concentration of PCB-153, the major congener, among different studies, it ranged from 30 to 140 ng/g lipid (Longnecker et al. 2003) in general populations from the United States, the Netherlands, Germany, and Canada, and in our study the concentration was 140 ng/g lipids. Considering half-lives of PCBs and the general decline in body burdens in most parts of the world (Cerna et al. 2007; Noren and Meironyte 2000), and noting the fact that the specimens from the Netherlands study were measured in the early 1990s, the residents in eastern Slovakia are currently exposed to high levels of PCBs.

The clinical implication of this reduced thymic index remains to be clarified. Nonetheless, the reduction in thymic index associated with an increase across the interquartile range of PCBs was comparable to the reduction associated with a 1-week premature delivery: both were −3%. Thus, the magnitude of the impact of this level of PCBs might be equivalent to a one-week delay in thymic maturation, although the actual mechanism and consequences are unknown. Because we are following the children in this birth cohort, in future analyses, we will examine longitudinal data for thymic index measured at 6 and 16 months to determine whether prenatal or postnatal PCB exposures influence the trajectory of this organ, and to evaluate associations of PCBs with other immune parameters as well as with clinical outcomes.

## Figures and Tables

**Table 1:** Characteristics of the study cohort consisting of 982 mother–infant pairs with deliveries 2002–2004 in two districts of eastern Slovakia.

Characteristic	No. (%)	Characteristic	No. (%)
District		Maternal smoking	
Michalovce	702 (71.5)	No	613 (62.4)
Svidnik	280 (28.5)	Yes	357 (36.4)
Maternal age (years)		Missing	12 (1.2)
≤19	84 (8.6)	Maternal alcohol consumption	
20–29	689 (70.1)	No	673 (68.5)
≥30	209 (21.3)	Yes	298 (30.4)
Maternal education		Missing	11 (1.1)
Basic schooling	204 (20.8)	Parity	
High school without graduation	260 (26.5)	0	404 (41.2)
High school with graduation	443 (45.1)	1	328 (33.4)
More than college/university	72 (7.3)	2	171 (17.4)
Missing	3 (0.3)	3	75 (7.6)
Sex of child		4	2 (0.2)
Male	503 (51.2)	Missing	2 (0.2)
Female	479 (48.8)	Maternal allergy history	
Ethnicity		No	937 (95.4)
Slovakian/other eastern European	757 (77.1)	Yes	35 (3.6)
Romani	215 (21.9)	Missing	10 (1.0)
Missing	10 (1.0)	Maternal asthma history	
Marital status		No	973 (99.1)
Married or living with partner	905 (92.2)	Yes	9 (0.9)
Never married	59 (6.0)	Maternal respiratory history	
Divorced	7 (0.7)	No	774 (78.8)
Missing	11 (1.1)	Yes	198 (20.2)
		Missing	10 (1.0)

**Table 2:** Univariate distribution of thymic index by six radiologists.

	Radiologists					
	Michalovce				Svidnik	
	1	2	3	4	5	6
No.	363	155	89	95	187	93
Mean	8.1	8.7	8.3	8.1	12.8	13.2
Median	7.6	8.3	8.0	8.0	11.6	13.2
SD	3.3	3.0	2.7	2.8	5.3	4.2
10th–90th	4.5–12.2	5.0–12.3	5.3–11.0	4.8–12.3	7.3–20.2	8.3–18.5

**Table 3:** Final multiple linear regression model of log thymic index with log PCBs and other covariates as predictors (*n* = 982).

Predictor variable	β	SE	*p*-Value
PCB [natural log-transformed (ng/mg serum lipids)]^a^	−0.036	0.018	0.047
Sex (male vs. female)	−0.102	0.023	< 0.0001
Smoking	−0.032	0.024	0.181
Alcohol consumption	−0.051	0.026	0.050
Ethnicity (Romani vs. Slovakian/other Eastern European)	0.104	0.030	< 0.001
Gestational age at delivery (weeks)	0.031	0.010	0.003
*Z*-score of birth weight	0.126	0.012	< 0.0001
District (Michalovce vs. Svidnik)	0.417	0.031	< 0.0001
Respiratory infection histories	−0.066	0.029	0.023

a For units of ng/g, this coefficient is multiplied by 10^−3^.

**Table 4:** Final multiple linear regression model of log thymic index for the most experienced radiologist, no. 5 from Svidnik, with log PCBs and other covariates as predictors (*n* = 187).

Predictor variable	β	SE	*p*-Value
PCB [natural log-transformed (ng/mg serum lipids)]^a^	−0.102	0.047	0.033
Sex (male vs. female)	−0.097	0.058	0.100
Smoking	−0.067	0.060	0.263
Alcohol consumption	−0.085	0.059	0.153
Ethnicity (Romani vs. Slovakian/other Eastern European)	0.135	0.079	0.092
Gestational age at delivery (weeks)	0.053	0.023	0.022
*Z*-score of birth weight	0.113	0.032	< 0.001
Respiratory infection histories	−0.181	0.090	0.046

a For units of ng/g, this coefficient is multiplied by 10^−3^.
